# 3D collagen type I matrix inhibits the antimigratory effect of doxorubicin

**DOI:** 10.1186/1475-2867-10-26

**Published:** 2010-08-13

**Authors:** Emilie Millerot-Serrurot, Marie Guilbert, Nicolas Fourré, Wojciech Witkowski, Georges Said, Laurence Van Gulick, Christine Terryn, Jean-Marie Zahm, Roselyne Garnotel, Pierre Jeannesson

**Affiliations:** 1 UMR CNRS/URCA n°6237, UFR Pharmacie, 51 rue Cognacq-Jay, 51096 Reims Cedex, France; 2 IRI - CNRS USR3078, Parc de la Haute Borne, 50 Avenue Halley, 59650 Villeneuve d'Ascq Cedex, France; 3 Plateforme Imagerie Cellulaire et Tissulaire, IFR 53, 51 rue Cognacq-Jay, 51096 Reims Cedex, France; 4 INSERM UMRS 903, CHU Maison Blanche, 45 rue Cognacq-Jay, 51092 Reims Cedex, France

## Abstract

**Background:**

The cell microenvironment, especially extracellular matrix proteins, plays an important role in tumor cell response to chemotherapeutic drugs. The present study was designed to investigate whether this microenvironment can influence the antimigratory effect of an anthracycline drug, doxorubicin, when tumor cells are grown in a matrix of type I collagen, a three-dimensional (3D) context which simulates a natural microenvironment.

**Methods:**

To this purpose, we studied the migratory parameters, the integrin expression, and the activation state of focal adhesion kinase (FAK) and GTPase RhoA involved in the formation of focal adhesions and cell movement. These parameters were evaluated at non toxic concentrations which did not affect HT1080 cell proliferation.

**Results:**

We show that while doxorubicin decreased cell migration properties by 70% in conventional two-dimensional (2D) culture, this effect was completely abolished in a 3D one. Regarding the impact of doxorubicin on the focal adhesion complexes, unlike in 2D systems, the data indicated that the drug neither affected β1 integrin expression nor the state of phosphorylation of FAK and RhoA.

**Conclusion:**

This study suggests the lack of antiinvasive effect of doxorubicin in a 3D environment which is generally considered to better mimic the phenotypic behaviour of cells *in vivo*. Consistent with the previously shown resistance to the cytotoxic effect in a 3D context, our results highlight the importance of the matrix configuration on the tumor cell response to antiinvasive drugs.

## Background

Cancer metastases are particularly challenging for the medical field and represent a major hurdle in cancer chemotherapy of solid neoplasia. This process confers poor prognosis for the affected patient. In fact, it is generally considered that 90% of human cancer deaths can be attributed to local invasion and/or distant metastases [1]. Given the fact that most current chemotherapeutic protocols aim at direct inhibition of tumor cell growth, the development of antimetastatic agents could be an effective means to prevent colonization, thereby enabling the containment of primary tumors in a chemically manageable form [2]. In the search of antitumor agents with anti-invasive properties, evidence has recently accumulated indicating that anthracyclines, one of the most potent classes of chemotherapeutic agents in clinical use, inhibit tumor cell invasion [3]. Indeed, doxorubicin [4,5] and related compounds such as aclacinomycin [6], or DA-125, a doxorubicin analogue [7], have been shown to inhibit *in vitro *invasion of various tumor cell lines originating from solid tumors when used at low concentrations. The underlying mechanism deals with either downregulation of matrix metalloproteinases [7] or disorganization of cytoskeleton and focal contacts [8].

One of the limitations of such studies is that they were performed on conventional tissue culture substrate, a situation that does not take into account the tumor cell microenvironment in which tumor cells *in vivo *exert complex interactions with their immediate neighbors and the extracellular matrix (ECM). For this reason, it is of primary importance in the search of cell invasion inhibitors to consider the tumor cell microenvironment in *in vitro *drug screening regimens. Indeed, it has been demonstrated as a key determinant of cell response to anticancer drugs; this, being due to multiple mechanisms including limited drug penetration, tumor cell adaptation to hypoxia, presence of an acidic extracellular pH and direct contact between cancer cells and the extracellular matrix or adjacent cells that induce cell-mediated adhesion resistance [9]. *In vivo*, such environmental causes have been suspected to contribute to cancer cell survival after initial therapy, allowing resistant cells to proliferate.

To evaluate the impact of microenvironment on the drug response, different types of matrices have been studied such as classical two-dimensional (2D) matrices or more recently fibroblast-derived three-dimensional (3D) matrices [10,11]. Studies have mainly focused on the cytotoxic or proapoptotic effects induced by an extensive panel of well-known chemotherapeutics either microtubule-disturbing agents such as vinca-alcaloids, taxoids or DNA-damaging compounds such as nitrogen mustards and anthracyclines [12,13]. It has been shown that epithelial and leukemic cell lines when grown onto such matrices may respond differently to these drugs and in some cases acquire cell mediated-adhesion resistance [10]. In accordance to this, we recently reported such a microenvironment-dependent resistance for the anti-invasive effect of anthracyclines. Indeed by contrast to plastic, human fibrosarcoma cells cultured on 2D surfaces coated with ECM proteins collagen type I and fibronectin were not affected by the antimigratory effect of doxorubicin [4]. Compared to evaluation of drug responsiveness of cells cultured on 2D coatings or onto 3D matrices, culturing cells within 3D matrices offers both a more realistic view due to the coupling of chemical and mechanical signals that takes place in the real tissues and better simulates cell response to antitumor drugs [14]. Since ECM appears as an important regulator of tumor cell response to chemotherapeutics and since nothing is known about the anti-invasive effects of anthracyclines in a 3D context, this study was designed to investigate whether the cell microenvironment can influence the antimigratory effect of the anti-tumor drug doxorubicin when human fibrosarcoma HT1080 cells are grown in a 3D collagen type I matrix. To this purpose, the current study examined migratory parameters by time-lapse videomicroscopy, the actin cytoskeleton organisation and the integrin expression. The activation state of FAK [15] and GTPase RhoA [16], two proteins involved in the formation of focal adhesion complexes and cell movement were also investigated. The data show that this 3D context protects the tumor cells against the antimigratory effect of non cytotoxic doses of doxorubicin.

## Results

### Determination of doxorubicin subtoxic concentrations in 3D cultures

**Figure 1:**
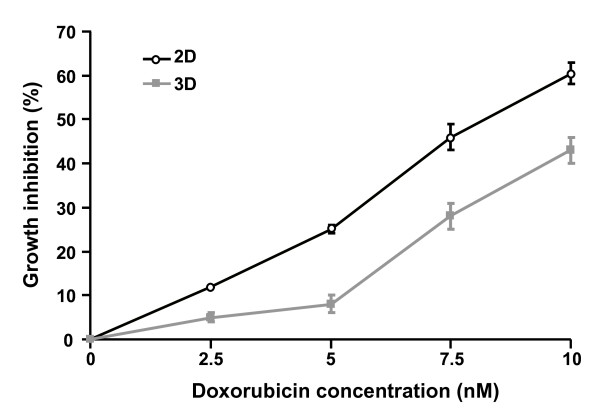
**Antiproliferative effect of doxorubicin in HT1080 cells cultured within 3D matrix**. After 48 h of culture in 3D matrix or on 2D plastic, cells were treated with doxorubicin at 0, 2.5, 5, 7.5 or 10 nM for 24 h. Then, cells were counted to evaluate cell number. Values represent the mean ± S.E.M. of three independent experiments.

To rule out a putative direct cytotoxic impact of the drug on cell migration, the ability of doxorubicin to decrease tumor cell motility was investigated at concentrations exhibiting no or limited effect on tumor cell proliferation. To this end, the effect of increasing concentrations of doxorubicin (up to 10 nM) on HT1080 cell growth was determined on cells either cultured in 3D collagen type I matrices or cultured on 2D plastic substrata. Figure 1 indicates that doxorubicin at 2.5 and 5 nM inhibited 3D cell proliferation by less than 10% when compared with the control. By contrast, for 7.5 and 10 nM, cells experienced a significant increase in antiproliferative effect by 28% and 43%, respectively. On 2D plastic, although more marked growth inhibitory effects were observed for the same drug concentrations, this inhibition remained limited for 2.5 and 5 nM which inhibited cell growth by 12 and 25% respectively. For both types of culture, cell viability was not affected. Taking into account these results, the impact of doxorubicin on 3D cell migration was studied in all further experiments at concentrations of 2.5 and 5 nM, which did not significantly influence cell proliferation, and at 7.5 nM as representative of a moderate cytostatic effect.

### Evaluation of the antimigratory effect of doxorubicin in 3D

**Figure 2:**
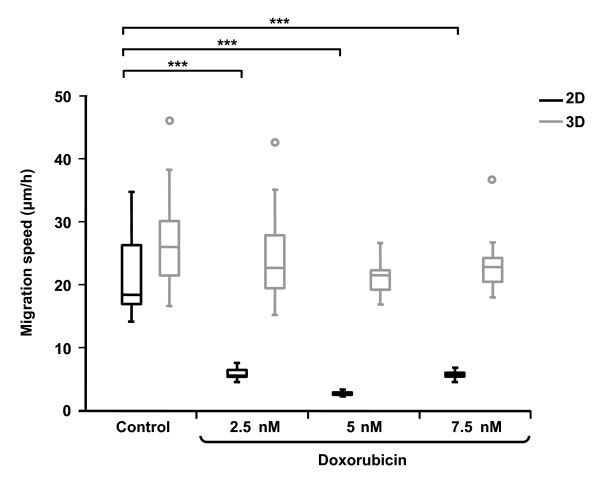
**Effect of doxorubicin on 3D HT1080 cell migration speed**. Cells cultured during 48 h within 3D matrix or on 2D plastic were incubated with doxorubicin at 0, 2.5, 5 and 7.5 nM for 24 h and were tracked by time-lapse videomicroscopy during the last 12 h of drug incubation. The migration speed was displayed as a box plots (bottom of box, 25th percentile; and top of box, 75th percentile) and middle line indicates the median (15 cells per sample). *** p < 0.001 significant difference between control and doxorubicin treatment.

**Figure 3:**
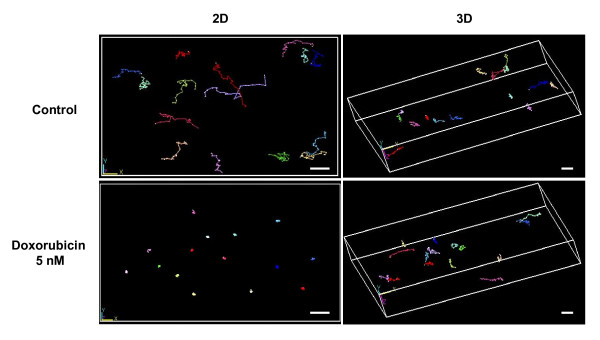
**Quantitative analysis of 3D trajectories on doxorubicin-treated HT1080 cells**. Cells cultured during 48 h within 3D matrix or on 2D plastic were incubated with doxorubicin 0 and 5 nM for 24 h and were tracked by videomicroscopy during the last 12 h of drug incubation. Each colour on the figure corresponds to different cells (15 cells per sample). (Bar = 100 μm).

The migration ability of HT1080 cells cultured within collagen type I matrices has been determined by quantifying their migration speed and their 3D trajectories using computer-assisted videomicroscopy. To this end, HT1080 cells were incubated with doxorubicin and individual cells were examined for their migrating potential between 12 h and 24 h after incubation with the drug. As previously shown on 2D plastic [4], doxorubicin induced a significant antimigratory effect at the non cytotoxic doses of 2.5 and 5 nM (Figure 2). Indeed, treated cells exhibited a median speed of 5 μm/h compared to 18 μm/h in control cells, indicating that the drug inhibited cell migration by about 70%. By contrast, for the same drug concentrations, the 3D collagen type I matrix totally abolished this drug antimigratory effect since the median speed of treated cells (23 μm/h) is similar to that of control cells (25 μm/h). This 3D collagen protective effect was also observed for the low cytostatic concentration of 7.5 nM. In addition to the estimation of the migration speed, continuous single cell tracking permitted the investigation of trajectories of cells grown in both models and treated or not with doxorubicin 5 nM (Figure 3). In 2D, whereas control cells moved along linear paths, treated cells exhibited circular trajectories around their starting point, in accordance with cell migration speed inhibition. By contrast, in 3D, the trajectories of treated cells appeared similar to that of control cells, which is in agreement with the protective effect of collagen on cell migration speed. Taken together, our data indicate that the 3D collagen type I matrix protects tumor cells against the antimigratory effect of doxorubicin.

### Cell phenotype in 3D and impact of doxorubicin

**Figure 4:**
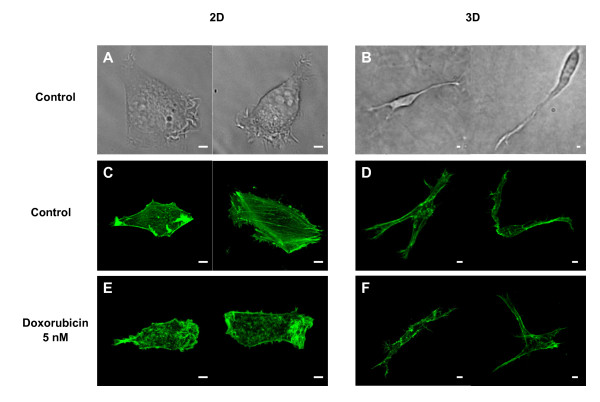
**Analysis of doxorubicin impact on 3D HT1080 cell morphology and actin cytoskeleton**. Cells cultured during 48 h within 3D type I collagen matrix or on 2D substrata were incubated with doxorubicin 0 and 5 nM for 24 h. Typical morphology of cells seeded on 2D substrata (**A**) or cultured within 3D matrix (**B**) were obtained by phase contrast microscopy. F-actin was stained by immunofluorescence with Alexa Fluor 488 phalloidin and captured using confocal microscopy in control cells in 2D (**C**) or 3D (**D**), and in treated cells in 2D (**E**) or 3D (**F**). (Bar = 5 μm).

Inside the cell matrix, HT1080 cells adopted a morphology dramatically distinct from the one observed in cells growing on planar substrata as shown by phase contrast microscopy and localization of F-actin (Figure 4). In 2D, cells develop a flattened morphology with a classical tear-drop shape displaying a wide leading edge and a narrow tail (Figure 4A). By contrast, cells in 3D matrices demonstrate extremely elongated cell bodies with a far more compacted nucleus which can be bipolar or stellate (Figure 4B). Cylindrical branched pseudopodia structures are also observed during active phases of migration when cells explore the microenvironment to find paths between the collagen fibrils (additional files 1 and 2). Compared to 2D, where cells exhibit a dense and regular actin stress fiber network (Figure 4C, additional files 3 and 4), in 3D models cells show some actin bundles in the cell body continuing into pseudopodia; actin patches were also found near the cell membrane (Figure 4D, additional files 5 and 6). In addition, there was no significant difference in the total amount of actin present between 3D and 2D grown cells (74 ± 12 arbitrary units/cell in 3D vs 87 ± 11 arbitrary units/cell in 2D, n = 10 cells). In 2D, treatment with 5 nM doxorubicin induces a loss of cell polarity and a dramatic disorganization of the stress fibers (Figure 4E, additional files 7 and 8) whereas in 3D, cell morphology and actin distribution were similar to that of control cells (Figure 4F, additional files 9 and 10). These results clearly demonstrate that in 3D, non toxic concentrations of doxorubicin have no effect on cytoskeleton integrity which is consistent with the collagen protective effect on cell parameters.

### Effect of doxorubicin on migration molecular regulators in 3D

**Figure 5:**
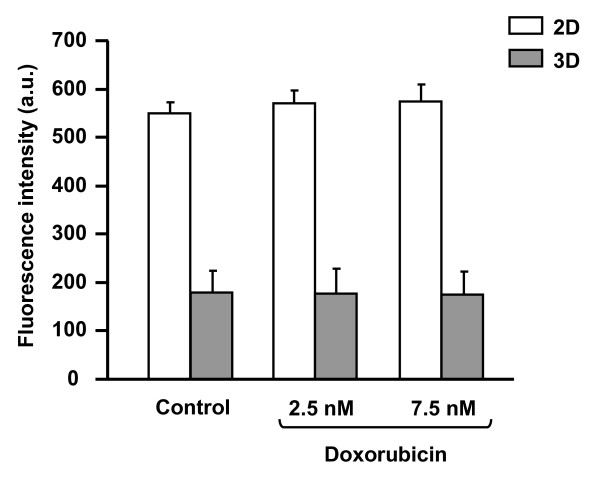
**Effect of doxorubicin on β1 integrin expression in 3D HT1080 cells**. Cells cultured during 48 h within 3D type I collagen matrix or on 2D substrata were incubated with doxorubicin 0 and 5 nM for 24 h. Then, they were stained with total-β1 integrin monoclonal antibody and analyzed by flow cytometry. Data shown are representative of three independent experiments.

**Figure 6:**
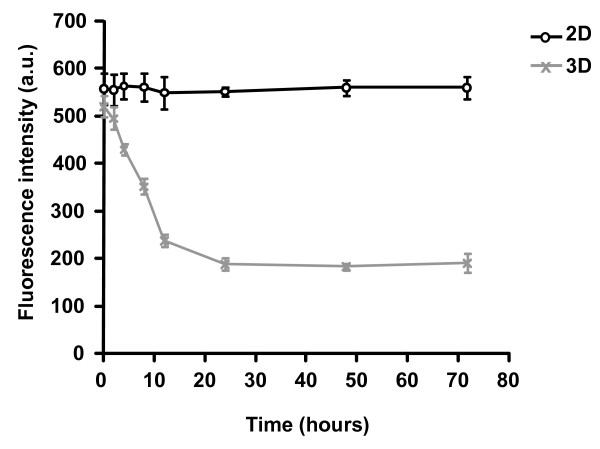
**Differential expression of β1 integrins in 2D and 3D HT1080 cells**. Cells were cultured in 3D collagen gel or on 2D substrata and flow cytometric analysis of β1 integrins was performed up to 72 hours. Data shown are representative of three independent experiments.

**Figure 7:**
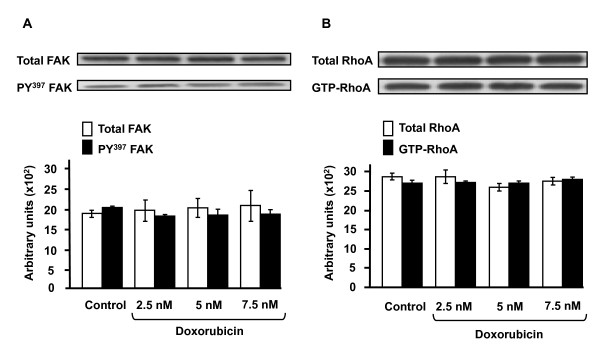
**Effect of doxorubicin on FAK and RhoA expression in 3D HT1080 cells**. Cells were cultured during 48 h in 3D collagen gel then treated with 2.5, 5 or 7.5 nM of doxorubicin for 24 h. Representative immunoblot of total FAK or Tyr^397^-FAK (**A**) and of total RhoA and RhoA-GTP (**B**). The graphs show the mean values of three independent experiments.

Since in 2D tumor cell motility is strongly dependent on the expression and functioning of integrins, FAK [15] and RhoA [16], these proteins were analyzed in 3D after drug treatment. As shown by flow cytometry (Figure 5), expression of β1 integrins, which are representative of the integrins expressed in HT1080 cells, was found to be unaffected by doxorubicin at 2.5 and 7.5 nM in 3D. Similar results were obtained with the same concentrations of drug in 2D culture suggesting that doxorubicin does not affect the expression level of integrins in presence or not of matrix proteins. In addition, it has to be noticed that in 3D culture the level of β1 integrin expression is 3-fold lower than in 2D culture. This decrease takes place during the first 10 hours of 3D culture and is in agreement with the dramatic morphological changes observed in 3D cells (Figure 6). Figure 7 depicts the western blot analysis of FAK (Figure 7A) and RhoA (Figure 7B) in 3D cultures treated or not with 2.5, 5 and 7.5 nM of doxorubicin. The data indicate that neither expression nor activation of these two proteins was influenced by the drug. These results strongly contrast with those obtained in 2D plastic cultures where the drug dramatically decreases the activation state of FAK and RhoA [4].

## Discussion

We demonstrate here that 3D type I collagen matrix protects human fibrosarcoma cells against the antimigratory effect of subtoxic concentrations of the antitumor drug doxorubicin. To our knowledge, this is the first study to show that ECM proteins in a 3D configuration are able to protect cancer cells from the antimigratory effect of a drug. This protective effect can be assimilated to a new form of environment-mediated drug resistance recently described for the cytotoxic effect of various antitumor drugs [9].

The effect of doxorubicin on tumor cell migration was studied here at concentrations moderately affecting or not cell proliferation in order to exclude the possibility that its antimigratory effect could result from a non specific consequence of the cytotoxic effect of this drug. Moreover, this is of considerable interest since the use of doxorubicin is often limited by severe cardiotoxicity and other undesirable side effects [17]. The 3D culture system used here is a 3D gel of type I collagen, which corresponds to a representative model that mimicks *in vivo *microenvironment. Type I collagen is a main component of extracellular matrix in the body connective tissues, through which tumor cells usually move to form metastases, and use as a pre-intravasation microenvironment [10]. The 3D collagen gel prepared here at physiological concentration [18] approximates the 3D fibrous nature of a mesenchymal stroma by displaying a highly fibrillar organization as shown previously [19]. In addition, due to its preparation without pepsinization, this native fibrillar collagen presents intact telopeptides in contrast with most of the experimentally generated collagen matrices currently using pepsin-cleaved collagen type I [20]. Telopeptides which correspond to the flanking regions of the molecules permit to form intra- and intermolecular cross-links that promote the staggering and the resilience of fibrillar collagen [21]. Such a 3D culture model has previously been shown not to affect doxorubicin penetration both in the gel and the tumor cells, indicating that the subtoxic concentrations defined in 2D can be easily transposed to 3D conditions. Indeed, by using quantitative microspectrofluorimetry, it has been shown that doxorubicin rapidly diffuses through the 3D collagen gel so that, after 1 h of incubation, drug concentrations are similar in the 3D matrix and the medium covering the gel. In addition, the intracellular penetration of doxorubicin in 3D cells is not modified compared to 2D conditions [19].

The behaviour of HT1080 cells cultured in the 3D matrices appears dramatically different from that of cells cultured on rigid and planar 2D substrata. Indeed, on 2D, cells exhibit the classical tear-drop morphology with a flat cross-section and a broad lamellipodium. On the other hand, in 3D, they adopt a bipolar, spindle or stellate shape, and express a lower level of β1 integrins which can be explained by the marked decrease of their cell volume as estimated by flow cytometry (data not shown). The observed cell protrusions constituted of thin and cylindric dendritic extensions are in favour of a mesenchymal migration mode [22]. As recently proposed [19], and as shown in our videomicroscopy sequences, these protrusions may sense the surrounding matrix and those that extend in a favourable direction then guide cell movement. Whereas HT1080 cells migrating across 2D plastic surfaces present classical actin stress fibers, those moving through 3D collagen lack actin stress fibers but exhibit a cortical cytoskeleton and actin-rich tips in their dendritic extensions. Such structural changes are similar to those observed for human foreskin fibroblasts seeded on top of relaxed type I collagen matrices [23] and appeared to be a characteristic of the cell-collagen matrix interface. This high degree of plasticity in cell shape and actin organization may be particularly favourable during the metastasis process and may contribute to the high invasiveness potential of this cell line in animal models [24].

Cell migration speed and individual trajectories were determined using time-lapse videomicroscopy and interactive cell tracking in a four-dimensional data set [25]. It is demonstrated that cell migration speed is higher in 3D compared to plastic substrate (25 μm/h *versus *18 μm/h, p < 0.01 data not shown). In 2D, doxorubicin markedly slows down cell locomotion by decreasing cell migration speed by about 70% and by disorganizing actin cytoskeleton. Our data are in accordance with previous studies demonstrating that other derivatives of anthracycline drugs, such as aclacinomycin [6] or DA-125 [7], also exhibited a significant antimigratory effect on plastic. Surprisingly, in 3D collagen matrix, this antimigratory effect was completely abolished as proved by the absence of drug impact on cell migration speed and cytoskeleton organization. The fact that the global content of actin is roughly the same in 2D and 3D grown cells, rules out the assumption that doxorubicin has no pharmacological target (i.e., actin), thus no impact in 3D culture. Such data are completely in agreement with the inhibitory effect of ECM components on the cytotoxic mechanism of various antitumor drugs [26]. This was demonstrated with cell lines originating from hematopoietic or solid tumors. Indeed, these cells are protected from drug-induced apoptosis when cells were plated on ECM proteins such as fibronectin, laminin-1 and collagens [27]. In addition, our results also show that the activation of both FAK and GTPase RhoA, key regulator enzymes of cell motility, that markedly decreased in 2D by doxorubicin [4], is unaffected in 3D. These data are in agreement with the recent demonstration that biopsies of doxorubicin-treated breast cancer patients, exhibited upregulation of genes involved in the focal adhesion pathway [28]. *In vivo*, ECM proteins such as collagen type I may conceivably impair the pharmacological properties of anthracyclines, triggering an intrinsic chemotherapeutic resistance. In addition, the development of selective inhibitors of FAK is proposed as a promising way of inhibiting migration and invasion [29]; the present results provide substantial evidence that the microenvironment parameter has to be considered to isolate such inhibitors.

## Conclusion

Our data clearly demonstrate that ECM proteins inhibit the antimigratory effect of the antitumor drug doxorubicin. They support the crucial role of the tumor environment in the failure of clinical response to chemotherapeutic agents and the emergence of an environment-mediated drug resistance. This experimental approach should be taken into account for a better definition of robust *in vitro *drug screening tests, this in order to find new agents specifically targeting the *in vivo *migrating behaviour of tumor cells.

## Methods

### Cell line

The human fibrosarcoma HT1080 cells (CCL-121) were purchased from the American Type Culture Collection (ATCC, Rockville, USA) and cultured in MEM with Earle salts and Glutamax I (Invitrogen, Cergy-Pontoise, France) supplemented with 10% fetal bovine serum (FBS) (Invitrogen). Cultures were maintained at 37°C in a humidified atmosphere containing 5% (v/v) CO_2_. Cells were routinely passaged at preconfluency using 0.05% trypsin-0.53 mM EDTA (Invitrogen) and screened for the detection of mycoplasma using PCR methods.

### 3D culture and drug treatment

Acid-extracted, non-pepsinized collagen type I from rat tail tendons was prepared as already described [30]. Lyophilized collagen was dissolved in sterile 18 mM acetic acid at a concentration of 3 mg/ml. To prepare 3D cultures, 5 × 10^4 ^HT1080 cells were resuspended in 50 μL FBS and mixed with a solution containing 50 μl MEM 10× (Sigma-Aldrich, L'Isle d'Abeau Chenes, France), 50 μl NaHCO_3 _0.26 M, 50 μl H_2_O, 45 μl NaOH 0.1 M, 5 μl glutamine 200 mM and 250 μl collagen 3 mg/ml. This solution was deposited in 24-well plates (500 μl/well). After polymerization at 37°C during 10 min, gels were recovered by 500 μl MEM 10% foetal calf serum and 3D cultures were incubated during 48 h before drug treatment. Then, medium recovering 3D cultures was replaced by 500 μl of fresh complete culture medium containing or not 2-fold concentrations of doxorubicin (TEVA Pharma S.A., Courbevoie, France). After 24 h, gels were digested by collagenase P at 1 mg/ml (Roche, Meylan, France), cell viability and cell number were determined by phase contrast microscopy. In parallel, conventional 2D cultures were performed by seeding HT1080 cells in 24-well plates at a concentration of 2 × 10^4 ^cells/ml (1 ml/well). After 48 h, culture medium was removed and cells were exposed to the doxorubicin treatment for 24 h.

### 3D cell migration quantification and actin staining

3D and 2D cultures, prepared as described above, were seeded in 12-well plates (2 ml/well). Cell motility was analyzed by time-lapse videomicroscopy using an inverted microscope Axiovert 200 M (Zeiss, Le Pecq, France) equipped with a small transparent environmental chamber Climabox (Zeiss) with 5% (v/v) CO_2 _in air at 37°C. The microscope was driven by the Metamorph software (Roper Scientific, Evry, France) and images were recorded with a charge-coupled device camera CoolsnapHQ (Roper scientific). Cell migration was quantified using an interactive tracking method as previously described [25]. For actin staining, after 24 h of incubation in presence of drug, cells were fixed using 4% paraformaldehyde in PBS for 1 h at room temperature. Cells were permeabilized with 0.5% Triton X-100 in PBS for 5 min. Samples were blocked with PBS containing 3% BSA with 0.3% Triton X-100 for 45 min. F-actin was detected using Alexa Fluor 488 Phalloidin (1:500, Invitrogen). Fluorescence images were captured using the confocal laser scanning microscope Zeiss LSM 710 (Zeiss). The actin content of the cells was quantitated by measuring the pixel fluorescence intensity using the ImageJ software [31].

### Flow cytometry of β1 integrins

After collagenase P treatment of 3D cultures or trypsination of 2D cultures, cells were incubated at room temperature for 45 min with PBS containing 10 μl antibodies against integrin subunits β1 coupled with FITC (Beckman Coulter, Villepinte, France) or 50 μl mouse IgG1-FITC as negative control (Beckman Coulter). Cells were then washed in PBS (pH 7.2) and assessed for fluorescence in a FACSCalibur flow cytometer (BD Biosciences, Le Pont de Claix, France).

### FAK activation assay

After collagenase P treatment, cells were lyzed with extraction buffer (Tris 10 mM pH 7.5, NaCl 150 mM, 1% (v/v) Triton X-100, 5% (v/v) PMSF 100 mM, 20% (v/v) NEM 30 mM, 20% (v/v) SDS 10%). Cell lysates were clarified by centrifugation at 10,000 g at 4°C for 10 min. Briefly, proteins were separated by 7% SDS-PAGE gels and transferred to a PVDF membrane. Then membranes were blocked with Tris-buffered saline (TBS) (20 mM Tris-HCl, pH 7.4, 137 mM NaCl) containing 0.1% tween (TBS-T) and 5% non-fat dry milk at room temperature during 1 h and incubated overnight at 4°C with either rabbit polyclonal antibodies raised against total FAK (1:5000, Millipore, Saint-Quentin en Yvelines, France) or [Y397] phosphorylated-FAK (1:1000, Millipore). Membranes were washed with TBS-T and incubated with peroxidase-conjugated anti-mouse IgG (1:20000, Millipore) at room temperature during 1 h. Chemiluminescent detection was realized by using an ECL^+ ^kit (GE Healthcare, Orsay, France).

### RhoA activation assay

To assess RhoA activation, the amount of RhoA-GTP bound to the Rhotekin RBD was determined using the Rho Activation Assay Kit (Cytoskeleton, Denver, USA) according to the manufacturer's instructions. Briefly, after collagenase P treatment of 3D cultures, cells were lyzed using the lysis buffer from the kit. Proteins were incubated with RBD-rhotekin beads for 60 min at 4°C. Beads were washed three times with wash buffer, resuspended in Laemmli sample buffer, and boiled for 2 min. Separation by 12% SDS-PAGE, transfer and blocking were processed as the FAK activation assay. Proteins were immunoblotted with anti-RhoA antibodies (1:500) and chemiluminescent detection was realized by using an ECL^+ ^kit (GE Healthcare).

### Statistical analysis

Data are presented as mean ± SEM except for migration speed which displayed as box-blot ranging from the 25th to 75th percentile including the median and whiskers from the 5th to 95th percentile. The values were analyzed with Kruskall-Wallis followed by Mann-Whitney test. Statistical significance was set at p < 0.05.

## List of abbreviations used

2D: two-dimensional; 3D: three-dimensional; BSA: bovine serum albumin; ECM: extracellular matrix; FAK: focal adhesion kinase; FBS: fetal bovine serum; MEM: minimum essential medium; PAGE: polyacrylamide gel electrophoresis; PBS: phosphate-buffered saline; TBS: tris-buffered saline.

## Competing interests

The authors declare that they have no competing interests.

## Authors' contributions

EMS, RG and PJ designed research. EMS, MG, NF, WW, GS performed research. LV, CT, JMZ contributed to analytic tools. EMS, MG, RG and PJ analyzed data and wrote the paper. All authors read and approved the final manuscript.

## Supplementary Material

Additional file 1 **Migration of HT1080 cells cultured in 2D**. Click here for file

Additional file 2 **Migration of HT1080 cells cultured within 3D collagen type I matrix**. Click here for file

Additional file 3 **Three-dimensional visualization of actin in HT1080 cells cultured in 2D**. Click here for file

Additional file 4 **Three-dimensional visualization of actin in HT1080 cells cultured in 2D**. Click here for file

Additional file 5 **Three-dimensional visualization of actin in HT1080 cells cultured within 3D collagen type I matrix**. Click here for file

Additional file 6 **Three-dimensional visualization of actin in HT1080 cells cultured within 3D collagen type I matrix**. Click here for file

Additional files 7 **Three-dimensional visualization of actin in HT1080 cells cultured in 2D and treated by doxorubicin 5 nM for 24 h**. Click here for file

Additional files 8 **Three-dimensional visualization of actin in HT1080 cells cultured in 2D and treated by doxorubicin 5 nM for 24 h**. Click here for file

Additional files 9 **Three-dimensional visualization of actin in HT1080 cells cultured within 3D collagen type I matrix and treated by doxorubicin 5 nM for 24 h**. Click here for file

Additional file 10 **Three-dimensional visualization of actin in HT1080 cells cultured within 3D collagen type I matrix and treated by doxorubicin 5 nM for 24 h**. Click here for file
